# Understanding the culture of antimicrobial prescribing in agriculture: a qualitative study of UK pig veterinary surgeons

**DOI:** 10.1093/jac/dkw300

**Published:** 2016-08-11

**Authors:** L. A. Coyne, S. M. Latham, N. J. Williams, S. Dawson, I. J. Donald, R. B. Pearson, R. F. Smith, G. L. Pinchbeck

**Affiliations:** 1 Department of Epidemiology and Population Health, Institute of Infection and Global Health, University of Liverpool, Leahurst Campus, Chester High Road, Neston CH64 7TE, UK; 2 School of Veterinary Science, Faculty of Health and Life Sciences, University of Liverpool, Leahurst Campus, Chester High Road, Neston CH64 7TE, UK; 3 Institute of Psychology, Health & Society, University of Liverpool, Bedford Street South, Liverpool L69 7ZA, UK; 4 The George Pig Practice, High Street, Malmesbury, Wiltshire SN16 9AU, UK

## Abstract

**Objectives:**

The use of antimicrobials in food-producing animals has been linked with the emergence of antimicrobial resistance in bacterial populations, with consequences for animal and public health. This study explored the underpinning drivers, motivators and reasoning behind prescribing decisions made by veterinary surgeons working in the UK pig industry.

**Methods:**

A qualitative interview study was conducted with 21 veterinary surgeons purposively selected from all UK pig veterinary surgeons. Thematic analysis was used to analyse transcripts.

**Results:**

Ensuring optimum pig health and welfare was described as a driver for antimicrobial use by many veterinary surgeons and was considered a professional and moral obligation. Veterinary surgeons also exhibited a strong sense of social responsibility over the need to ensure that antimicrobial use was responsible. A close relationship between management practices, health and economics was evident, with improvements in management commonly identified as being potential routes to reduce antimicrobial usage; however, these were not always considered economically viable. The relationship with clients was identified as being a source of professional stress for practitioners due to pressure from farmers requesting antimicrobial prescriptions, and concern over poor compliance of antimicrobial administration by some farmers.

**Conclusions:**

The drivers behind prescribing decisions by veterinary surgeons were complex and diverse. A combination of education, improving communication between veterinary surgeons and farmers, and changes in regulations, in farm management and in consumer/retailer demands may all be needed to ensure that antimicrobial prescribing is optimal and to achieve significant reductions in use.

## Introduction

Indiscriminate prescribing practices and the overuse of antimicrobials in food-producing animals have been implicated in the emergence of antimicrobial resistance in bacterial populations, with consequences for both animal and public health.^1,2^ The emergence of resistant infections in animal populations can impact health and productivity.^3^ Additionally there is concern over the potential for the zoonotic transfer of resistant bacteria and/or resistance genes, from livestock species to humans, which is a phenomenon recognized as a potential threat to human health through the use of antimicrobials in pigs.^4^ Whilst isolated incidents of such transfer are described in the literature,^5–7^ it is impossible to quantify or assess the level of the risk at present.^8^ Thus, it is essential that prudent antimicrobial practices are adopted in veterinary as well as in human medicine to minimize selection pressures with the aim of slowing the emergence of resistant bacteria.^9,10^

Antimicrobial use in pigs has been highlighted as an area of particular concern in the UK and Europe with the formation of working groups and research initiatives striving to ensure that use is responsible.^11–14^ Veterinary prescribing practices in the pig sector, such as the use of antimicrobials for disease prophylaxis,^11,15^ commonality of the administration of in-feed antimicrobials^11^ and relatively high sales of antimicrobial products authorized for use solely in pigs^14^ have highlighted them as a priority species in the UK and Europe for gaining a better understanding of prescribing and use.^11,16^

Following the 2006 EU ban on the use of antimicrobials for growth promotion, they are only permitted for use in therapeutic or prophylactic indications. Veterinary surgeons are the only professionals able to prescribe antimicrobials for veterinary use in the UK. A thorough understanding of veterinary surgeons’ current prescribing behaviours relating to antimicrobial regulation has been identified as being essential to the development of strategies to ensure that veterinary use is responsible.^16–18^

Antimicrobial prescribing does not happen in isolation, but takes place within an environment where factors both intrinsic and extrinsic to the prescriber influence decisions. In human medicine, factors intrinsic to the prescriber relate to their personal confidence in prescribing decisions, concern over the consequences of such decisions and attitudes surrounding a sense of responsibility to patients, whilst extrinsic factors are those external to and beyond the control of the prescriber, e.g. pressure from patients, other healthcare professionals and institutional policy.^19^ Whilst parallel intrinsic drivers have been identified in veterinary medicine,^20–22^ extrinsic pressures such as the financial viability and the influence of husbandry practices on prescribing decisions are unique to the role of livestock as food-producing animals.^17^ Therefore, it is necessary to understand how antimicrobial prescribing sits amongst other factors that might drive antimicrobial use.

There are a number of methodological approaches that can be taken to understand the relationship between behaviour, prescribing and antimicrobial use. Whilst some provide more structured empirical data, qualitative approaches are more appropriate in broad exploratory contexts such as antimicrobial resistance and veterinary prescribing.^23^ In this study we used in-depth qualitative interviews to explore the attitudes, motivations and reasoning behind prescribing decisions by pig veterinary surgeons. This is the only large-scale study of this type to be undertaken in the UK and provides novel insights into UK prescribing practices, which have potentially wide-ranging implications for the control of antimicrobial resistance and the economic landscape for pig farming.

## Methods

### Selection of participants

All veterinary practices listed as conducting pig work on the Royal College of Veterinary Surgeons veterinary practice database in England, Wales and Scotland^24^ were contacted by phone. Practices were asked to confirm that they still undertook some commercial pig work and if so the names of veterinary surgeons that treated pigs were requested. A final confirmed list of 261 veterinary surgeons and their 104 respective practices was made, which was believed to represent all practices that conducted commercial pig work. Data on the veterinary surgeons, such as gender and year and place of graduation were obtained from the Royal College of Veterinary Surgeon Registers. Participants were recruited using a purposive sampling technique, which aimed to obtain a sample population that contained a spectrum of veterinary surgeons working within the UK pig industry and thus included both male and female, partner and assistant, private practice and company practitioners with a range of levels of experience. Veterinary surgeons that had previously attended a focus group from a previous study on prescribing practice in pigs^25^ were excluded from the selection process.

All the interviewees were approached directly by telephone or e-mail to request participation and arrange a suitable time and location.

### Data collection

Qualitative in-depth interviews of a semi-structured nature were conducted. An interview guide was developed by the authors based on a review of the literature, current issues surrounding antimicrobial use and results from a previous focus group study exploring antimicrobial prescribing behaviours in pig veterinary surgeons and farmers.^25^ The interview guide consisted of open questions designed to encourage free and detailed discussion around influences on prescribing decisions and the use of antimicrobials in pigs and included the following key areas:

- views on the current debate over antibiotic use and antibiotic resistance;
- drivers and motivators of antibiotic prescribing and dispensing decisions;
- the licensing and legislation of antibiotics;
- husbandry practices and antibiotic use;
- antibiotic usage in the UK, EU and the rest of the world;
- barriers to reducing antibiotic use for both prophylaxis and treatment;
- the future of pig farming.

All interviews were undertaken by the author (L. A. C.) with an additional author (S. M. L.) also present for a number of interviews. Flexibility was allowed in the order and conduct of the interview and questions were phrased in an attempt to encourage participants to express their views and recount their stories. Further questions that arose during the interviews were phrased carefully to avoid leading the discussion.

Two pilot interviews were conducted and the interview guide was reviewed and revised by the authors. Transcripts from the pilot interviews were reviewed in detail by three of the authors and were considered to be of acceptable quality to include in the overall analysis. Interviews were conducted at a place and time convenient to the participant. Interviewees were given a participant information sheet that provided an overview of the project.

### Ethics

Permission to record the interviews was sought over the telephone when recruiting participants as it was considered a vital component of the qualitative interview process to facilitate the subsequent data analysis. All participants signed a consent form prior to the interview. Ethics approval for the study was gained from the University of Liverpool Veterinary Science Research Ethics Committee and the Department for Environment, Food and Rural Affairs survey control unit prior to commencing the study interviews.

### Thematic data analysis

The interview audio recordings were transcribed verbatim and anonymized. The transcripts were transferred into Atlas.ti V.7.7.1 (Atlas.to Scientific Software Development) for data management using a thematic approach. Despite being widely used in qualitative research, thematic analysis is poorly defined as a methodology, with approaches taken being diverse and sometimes variable.^26–28^ Thus, to ensure consistency of data analysis the six phase approach to thematic analysis described by Braun and Clarke was adopted.^26^ This approach has been widely used and accepted as being robust across a wide range of disciplines, including human health research.^29^

A theoretical approach to thematic analysis was used in which the coding of the transcript was motivated by the authors’ pre-existing coding frame from an earlier focus group study.^25^ Reiterative reading of the transcripts by two of the authors (L. A. C. and S. M. L.) was used to transform ideas generated into a set of codes to identify a feature of the data of interest to the researcher. These initial codes were then categorized into potential themes and coded data extracts within identified themes were reviewed and collated to form the minor themes. The two researchers conducting the data analysis concluded that data saturation was reached when no new descriptive codes or themes were identified from additional interview transcripts and therefore no further interviews were conducted once this had been achieved.

Themes were discussed and reviewed by a multidisciplinary team, including epidemiologists, veterinary surgeons and a researcher experienced in qualitative research methods to reflect on the relevance of the themes to the research questions.^30^ These themes were then refined to ensure that each was meaningful and clear but distinct from other themes.^31^ A thematic map was constructed to review the relationships between these minor themes. Minor themes that were linked by a common subject area or which related to an overall topic were grouped together, given a unique theme title and considered as major themes.

## Results

A total of 24 veterinary surgeons were contacted and 21 interviews were completed and included in the study (three practitioners were willing to take part in the study but it was not possible to arrange interviews due to geographic and time constraints). Table S1 (available as Supplementary data at *JAC* Online) gives an overview of the demographics of the sample population. All of the interviews were conducted by one or both of two authors (L. A. C. and S. M. L.) and lasted between 45 and 90 min.

**Figure 1.**
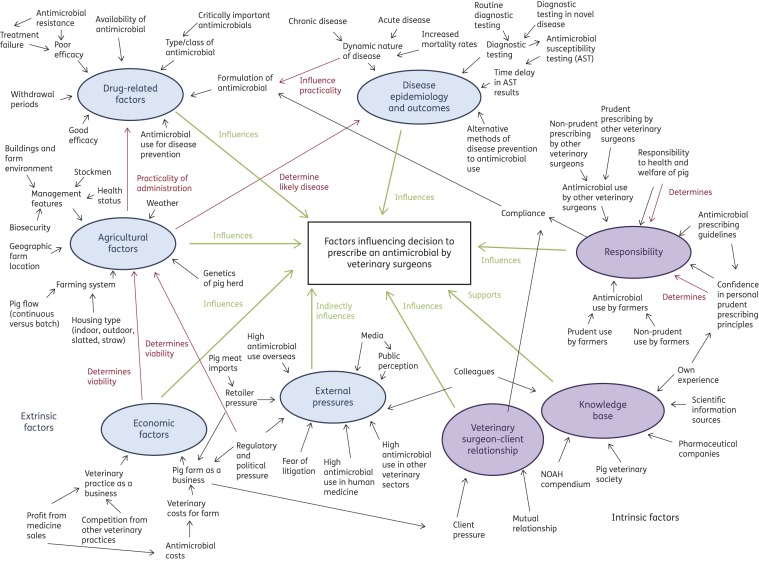
Thematic map demonstrating the relationship between minor and major themes. This figure appears in colour in the online version of *JAC* and in black and white in the print version of *JAC*.

Thematic analysis revealed eight major themes consisting of 76 minor themes that were identified as influencing antimicrobial prescribing behaviours. A thematic map is shown in Figure 1 and themes are shown in Table S2.

### Disease epidemiology and outcomes

In its most simplistic form, disease was considered a driver for antimicrobial prescription. Amongst interviewees, definitions of disease were diverse; on the one hand, participants defined disease in terms of distinct bacterial pathogens, such as *Salmonella* spp., *Streptococcus suis* and *Brachyspira hyodysenteriae*, whilst on the other hand, individuals proposed that disease syndromes consisted of multiple pathogens.

‘*… we're very often not treating singular pathogens. You're feeding a disease syndrome with multiple potential effects.*’

Whilst theoretically only bacterial disease should influence prescribing behaviours, most veterinary surgeons identified that this perception was too basic and perceived that viral disease played a role in prescribing behaviours. For example, when influenza virus infects a herd, antimicrobials may be used to treat the impact of secondary bacterial diseases.

‘*I think at finisher level [fattening pig near slaughter weight] the main antibiotics we would use for respiratory disease are used to control secondary bacterial infections … Flu is a massive issue. We all know you don't give antibiotics for flu. However, if you don't put in a course of CTC [Chlortetracycline] or something, pigs will then get something else and then you'll start seeing pigs die.*’

Disease was categorized as either chronic or acute by interviewees, with contrasting approaches to antimicrobial use and diagnostic testing in the two states. Routine diagnostic testing to monitor endemic and chronic disease levels was seldom cited as being the norm on most pig units. However, performing diagnostic testing when acute clinical signs were first seen was described as a common behaviour by participants, particularly when a novel pathogen or more severe form of an endemic disease was suspected. To ensure that pig welfare is not adversely affected, an antimicrobial must be selected based on a presumed diagnosis, observed on either clinical or post mortem examination before being able to confirm the choice through susceptibility profiles. This time delay in obtaining results from antimicrobial susceptibility testing (AST) was acknowledged as being problematic by veterinary surgeons. In parallel, the direct consequences of disease, morbidity and mortality, were considered to drive the use of antimicrobials for both therapeutic and prophylactic reasons.

‘*If you have animals that are acutely ill and are in the process of dying … you need to get antibiotics into them as quickly and as effectively as you can …*’

‘*That's why we always do culture and sensitivity on everything really. The very first day, if it's a severe disease, it would be a best guess on post-mortem as to what was causing the problem.*’

Some participants felt that endemic disease levels amongst the UK pig herd were high and that antimicrobials were used to reduce the impact of disease on production. Thus, antimicrobial use in these situations was considered aimed at managing disease levels on farms rather than on producing a clinical cure.

‘*The national herd is not very clean, and if you took antibiotics out tomorrow, bacterial disease would avalanche through the herd and the pig farm would become non-viable.*’

Vaccination was proposed by interviewees as an alternative method to antimicrobial use to prevent disease. The following quote bridged the major themes of ‘disease epidemiology and outcomes’ and ‘agricultural factors’, as it considers vaccination, health status and the facilities and management of a farm to be pivotal in minimizing the antimicrobial requirements of a farm.

‘*If you can get decent buildings, a decent stockman, decent health status, and you've got a reasonable vaccination programme in place to control any underlying health, then a lot of farms [would] manage with very little in-feed [antimicrobials medications?] …*’

### Agricultural factors

The sample population held a wide spectrum of opinions on which farming systems would be defined as higher and lower antimicrobial users. The farming systems considered included indoor units when compared with outdoor rearing and slatted-based pig accommodation in comparison to straw-based housing. Although opinions were diverse, there were a few dominant ideas. For instance, some participants identified that slatted systems were advantageous in their ability to minimize enteric disease on pig units, as pigs did not come into direct contact with faecal matter. A specific example given by some was that levels of *Salmonella* spp. were higher amongst straw-based pig units when compared with those that utilized slatted flooring.

‘*… we invented slatted systems so that we could separate the pig from its urine and its faeces. As soon as you separate a pig from its urine and faeces, they are infinitely healthier than before.*’

‘*Salmonella isolates from slaughter pigs … the solid floor finisher houses are the ones that have given us the biggest …*’

The majority of veterinary surgeons considered the quality of the unit management to be the most significant factor in avoiding the overuse of antimicrobials; a ‘well managed’ pig unit was thought to require fewer antimicrobials than one perceived to be managed poorly. Interviewees felt that having highly skilled stock people was pivotal to this with a few individuals making a direct link between having highly skilled staff and a minimal antimicrobial requirement on a unit, through early recognition of clinical disease signs and prompt therapeutic intervention.

‘*… the system absolutely influences diseases and therefore the use of antibiotics. Lower ones are always the ones that are well managed … if it is badly managed you can end up with problems and diseases so the management of each system is the key really.*’

‘*I think stockmanship is massive, it's seeing it, it's seeing a problem before it develops and getting in there because you may need less antibiotic use because we've caught something early.*’

Some management practices were considered to be limiting factors in reducing antimicrobial use on many farms. Participants linked the practice of mixing pigs from multiples sources, a continuous pig flow system and low health status herds with high disease burdens on pig units when compared with sourcing pigs from a single source, an all-in/all-out pig movement system and herds with a high health status. Additionally, interviewees also linked farm environment with antimicrobial use and considered old buildings with poorly maintained facilities to be higher antimicrobial users.

‘*The higher users* [*of antimicrobials*]*… would tend to be the older farms, longer established units, lower health status, continuous flow, poor hygiene, dubious management practices, and yes, a lack of attention to detail and management. Lower use would be high health units, perhaps more extensive, all-in, all-out by department or by batch, things like that.*’

### External pressures

Veterinary surgeons commonly cited poor public perception as a pressure on their professional lives. Two contrasting opinions were found amongst participants as to how the public perceived that pigs were produced. On the one hand, some participants proposed that the general public thought that a significant amount of antimicrobials were used in the pig industry and cited inaccurate media reporting as the grounding of this public opinion. Conversely, others held the opinion that the general public were unaware of the intensity of pig production and had an idealized image of smaller scale extensive agricultural systems with low antimicrobial use.

‘*I think in terms of food production … there is a perception that again pigs and poultry do get a lot of antibiotics. They'll believe what's on the front page of the tabloid papers and take it away, whether there's any proof or not.*’

‘*There is a complete disconnect … the general public has this vision of sort of utopian agriculture where a nineteen fifties Darling Buds of May farm with three pigs and ten chickens against the world, there is a complete disconnect between the two, the reality is cheap food on the shelves, intensive farming.*’

Veterinary surgeons voiced opinions on antimicrobial use in pigs overseas, in other veterinary sectors and in human medicine. The majority opinion amongst participants was that the pig veterinary sector was more responsible in its use of antimicrobials when compared with other species sectors. This view was more common amongst the specialist pig practitioners compared with individuals who worked in mixed species practice.

‘*I think that if you're looking for irresponsible use of antibiotics … you're probably looking at the wrong industry … the cattle guys and the sheep guys are much more difficult to get under control than the pig guys …*’

Many interviewees held the opinion that overprescribing is an issue in human medicine and some linked this to resistance in humans. Participants shared the opinion that issues of antimicrobial resistance in human medicine were predominantly driven by prescribing practices in humans and that any influence on human resistance profiles, from veterinary use, was negligible. Some participants expressed concern that the medical profession implicated the veterinary sector in resistance issues; this was identified as a pressure on prescribers.

‘*We know that medics have traditionally just dished out antibiotics to anybody.*’

‘*Personally, I suspect, and from things that I've read and seen, I suspect it's probably more the use of antibiotics in human medicine that has driven antibiotic resistance in the human bugs … rather than transfer from animals.*’

‘*… it's [antimicrobial resistance] obviously an issue in human medicine, which I think they're probably using us as the scape goats for. At the moment I think we've just got to be seen to conform or to reduce our usages to take the party line.*’

When compared on a global scale interviewees considered that the UK pig industry was a low antimicrobial user compared with many other countries. Many interviewees felt that antimicrobials were used in a greater volume and less responsibly overseas, which linked closely with the minor themes of importation pressure and retailer pressure. Participants felt that farmers were under pressure from retailers due to the high price of producing pig meat in the UK and the constant threat from supermarkets sourcing pig meat from abroad, where antimicrobial use is greater and prescribers face fewer regulations over prescribing.

‘*Go to America, go to Brazil, go to Thailand, all big players in moving multiple amounts of meat around the world and they are huge users of antimicrobials.*’

‘*There's no point at all in trying to hammer the UK farmer into using no antibiotic, for his product to become too expensive on the marketplace for the supermarkets to buy, and for the supermarkets then to buy in from countries that are still quite happy to dish out the antibiotic.*’

### Veterinary surgeon–client relationship

Client pressure was perceived to be a potential driver towards antimicrobial prescribing by veterinary prescribers. Most clients were considered to respect the decision of a veterinary surgeon, as to whether an antimicrobial was necessary or not; however, participants recognized a minority of ‘bullying clients’ that desired antimicrobials and applied pressure on veterinary surgeons to prescribe. The potential for blame should an antimicrobial not be prescribed, and later prove to be necessary, was identified by some interviewees as a specific factor in client pressure. An awareness of the potential for litigation under such circumstances was identified by a few participants as a possible driver for antimicrobial use.

‘*There are 75% of rational clients that you can discuss things with and reason as to why they don't need to use that antibiotic. And there is the 25% of damaging clients who will simply insist … that they have that …*’

‘*… there are some things that probably don't need antibiotics—almost certainly don't need antibiotics. But I think you're laying yourself open to litigation if you don't use them.*’

In contrast to the perception of client pressure, a minority view identified a relationship in which the veterinary surgeon and farmer worked together in a mutual partnership. This view was most commonly held either by practitioners who were partners or in a senior role within their respective practices. Participants generally placed the burden of responsibility for the prudent use of antimicrobials in pigs, on themselves, as the prescriber. However, some interpreted that the relationship between the veterinary surgeon and the farmer, resulted in a shared responsibility between both parties. These participants tended to acknowledge this mutual relationship much more commonly than those who considered the responsibility for prudent use to lie solely with the veterinary surgeon.

‘*With the vet and the doctor who's prescribing. They are the professionals, they should be able to see the bigger picture and it's their responsibility.*’

‘*… the farmer and the vet, together … working as a team … You can't pin it on one or the other; it's got to be both. Both need to be aware and willing to take it on board.*’

### Drug-related factors

Veterinary surgeons considered a complex interaction between different disease characteristics and the number of animals affected when deciding which formulation of antimicrobial to prescribe. In general, participants considered injectable antimicrobials were most commonly used in smaller groups of pigs whilst in-feed or in-water formulations were more appropriate for larger group sizes. Overall, interviewees preferred in-water medications in acute disease situations, as they can be administered more promptly than in-feed preparations. Conversely, in more chronic or ‘endemic’ disease situations the in-feed route was considered acceptable, as it offered the ‘cheapest’ and ‘easiest’ route of administration for farmers, when compared with in-water formulations.

‘*The sorts of thoughts that I would be thinking about would be … the number of pigs that you actually need to treat. Obviously you can't inject everything if it's a big group.*’

‘*[The] majority of the situations that you are dealing with would be that pigs would need immediate medication which you can only do with water because the feed cannot get there on time …*’

A general consensus across the interviews was that the use of in-feed antimicrobials for disease prevention was both commonplace and justifiable. The decision to continue or discontinue prophylactic in-feed antimicrobials was identified as problematic by many as there was concern that withdrawal may adversely affect pig welfare in situations where management improvements are either not economically viable or practically feasible. In addition, participants felt a sense of pressure from the reluctance of farmers to withdraw an antimicrobial perceived to be effective due to the high economic costs involved in discontinuing an antimicrobial and then being required to re-introduce it.

‘*I do believe in prophylactic treatments because there are too many times where you try and not use antibiotics and then you end up with a bad mortality [rate] …*’

‘*Some farmers are quite reluctant, I suppose, to pull out a lot of preventative medicines because if everything does go wrong it does cost them quite a bit.*’

The WHO categorized fluoroquinolones, third- and fourth-generation cephalosporins and macrolides as critically important antimicrobials of the highest priority for risk management in their use, to maintain efficacy for human use.^32^ Thus, the veterinary use of these highest priority critically important antimicrobials was an area for discussion in the interviews. Veterinary surgeons showed a great sense of awareness of this issue and recognized that the use of fluoroquinolones and third- and fourth-generation cephalosporins should be confined to clinical situations where no alternative antimicrobial is available. However, many described clinical examples of use, e.g. the use of a fluoroquinolone as a first-line treatment in neonatal scour. Participants justified this use due to the widespread resistance in diarrhoea pathogens to other antimicrobial classes and as alternative antimicrobial classes, that have been used historically, are either no longer marketed in the UK or no longer available.

‘*… the major use of fluoroquinolones is in piglet scours. If there was an alternative there then obviously, yes, we would use it … We just don't have something that's effective enough.*’

‘*… we lost neomycin and we were using neomycin quite a lot, so you know then we had to move on to something else, and that pushed us nearer to having to use fluoroquinolones than we'd like to go …*’

The majority of veterinary surgeons did not feel that resistant infections in pigs had a major impact on their clinical work despite commonly stating that neonatal scour pathogens frequently exhibited resistance on historic ASTs to multiple antimicrobial classes. Furthermore, the observation of treatment failures following the administration of an antimicrobial and the need to change to a different antimicrobial after initial treatment was a common theme, yet participants seldom linked this with antimicrobial resistance. Interviewees felt that it was difficult to identify a direct cause for these treatment failures; reasons commonly proposed included underlying viral disease challenge and incorrect or inefficient administration of antimicrobials.

‘*… sometimes it's difficult to know whether the treatment failure has been because of resistance to the antibiotic. Or whether it has been that the medicine hasn't been either administered or taken in by the animal correctly … If that's the case, you then try a different antibiotic, for the same problem, and you get a much better response.*’

The interviews gauged veterinary opinion on the potential impact should a restriction on the veterinary use of the critically important antimicrobials be implemented. There was a general consensus that the loss of third- and fourth-generation cephalosporins would have a minimal impact on the pig industry; however, there would be a negative impact should fluoroquinolones be restricted, and a greater effect should macrolides be included in a restricted list. Debate over the potential restriction of macrolides sparked strong views amongst veterinary surgeons; there was concern that a ban would have a major economic impact on the pig industry and would negatively affect pig welfare. A common example cited by interviewees was that if tylosin were no longer available for the treatment and control of *Lawsonia intracellularis*, which causes ileitis in pigs, then the cost of production would increase.

‘*… in terms of the fourth generation cephalosporin, I, as a veterinarian, try to avoid it like the plague …. The Fluoroquinolones … I think we would probably cope, but there are times when that's the only thing that will do, according to my diagnostics.*’

‘*Ileitis, while it's not a big problem in terms of mortality, does affect an awful lot of piglets …. I think the cost of production would increase as a result. We would also have more welfare issues; we'd have more tail bites; we'd have more agitation in pigs.*’

The meat withdrawal period on a product, which ensures that antimicrobial residues do not enter the human food chain, was identified as a driver in antimicrobial choice decisions by veterinary surgeons and was of particular importance when medicating pigs near slaughter. For example, an oral formulation of tylosin, a macrolide, was cited as being used frequently in pigs near slaughter due to its nil meat withhold.

‘*Tylosin is still an interesting antibiotic, because it has the nil withdrawal on it, which means it's the only thing that, if there is a lot of late onset pneumonia, you can actually put in, into pigs. But that's maybe more of an issue, that you could probably reduce the usage of Tylosin overnight by sticking a ten day withdrawal on it.*’

### Responsibility

Ensuring that optimum pig health and welfare is maintained was described as a driver for antimicrobial use by many veterinary surgeons and was considered a professional and moral obligation. There was a general consensus across the interviews that having a disease was the most important cause of poor welfare in pigs.

‘*People talk about welfare, they talk about how many pigs are in a pen or whether they've got something to play with in the early hours of the morning. Well actually, no, the most significant cause of any welfare problem to pigs is any disease that they may have.*’

‘*If I can just take one example. If we didn't use antibiotics in this one large … unit—I don't think we could continue, I really don't … The pigs’ welfare would be shot at as well. So although I know that it's the system, that's what we've got to work with. So therefore, they need the antibiotics.*’

In general, participants held the opinion that they considered themselves to use antimicrobials in a responsible manner, and that in general they would consider the pig sector to be similarly responsible. However, a minority of mixed species veterinary surgeons did not share this opinion. Issues raised by these participants included concern that there may be ‘overprescribing’ of antimicrobials within the pig sector and that the use of long courses of in-feed antimicrobials, for disease prevention, may not be prudent and are used as ‘management tools’.

‘*There are some that just really like to use—they want to throw everything at a problem. You wonder what you're writing the prescriptions out for. Antibiotics which have a little bit of food in or food which has a little bit of antibiotics in.*’

‘*I think the one [prescribing practice] that we as pig veterinarians are weak on are the habitual repeat users. It's the repeated in feed prescription that's the issue, isn't it? I'm as guilty as the next man of that.*’

Participants described conflict between their desire to limit the use of critically important antimicrobials as first-line choices and the desire to maximize farmer compliance. Some stated that they have used a critically important antimicrobial, due to the longer duration of action, to improve compliance and therefore ensure that the pig received the correct dose of antimicrobial. These participants identified that a long duration of activity would influence the choice of an antimicrobial as it would ensure higher compliance rates when compared with drugs that require repeat injections.

‘*… it's a conflict of interest between using the fourth generation, third generation cephalosporin as a second line treatment, which is ideal … with [using] a considered less important antibiotic for human health and the animal is not completing the course then …*’

‘*Certainly clients aren't keen to multi-jab. Yes, compliance is improved if you can say a single jab.*’

A minor theme that was infrequently encountered yet which sparked a very strong and passionate response was the notion that antimicrobials are still used, on occasion, for their beneficial effects on growth rates despite the 2006 EU ban on the use of antimicrobials for growth promotion. This minority opinion was held by mixed species practitioners rather than specialist pig practitioners. In the following quote, the veterinary surgeon proposed that the macrolide tylosin is used for its growth promotional properties rather than the prescribed indication, for the prevention of *L. intracellularis*:

‘*Tylan (tylosin) is a growth promoter. It is used as a growth promoter. There are thousands and thousands of tons of Tylan (tylosin) going in at relatively low rates. Whether you say it's against lawsonia, or whatever you call it, or whether you say it's growth … Ultimately, it's almost below a therapeutic … dose.*’

### Economic factors

Veterinary surgeons reported an awareness of the financial pressure that farmers were under and felt that this influenced antimicrobial prescribing decisions. Many participants felt driven to try to ‘reduce the cost’ of antimicrobials for farmers and did not feel that the profit margin on an antimicrobial would motivate prescribing behaviours. Thus, the majority felt that the ‘decoupling’ of antimicrobial sales, whereby veterinary surgeons are no longer able to dispense drugs directly to clients, would not affect the total amount of antimicrobials used in pigs.

‘*There is a margin on antimicrobials, and from the outside you can actually see that—it's rather bizarre, that this chap prescribes it and …supplies it. Is there a conflict? It genuinely doesn't enter into my prescribing decisions.*’

A minority felt that cost may motivate which antimicrobial a farmer requests from their veterinary surgeon and therefore proposed that increasing the price of critically important antimicrobials may result in fewer farmers requesting these drug classes.

‘*I've got a way to stop them using Fluoroquinolones, I actually make them expensive sometimes.*’

Antimicrobial use was considered by veterinary surgeons to be a short-term and less expensive solution to a disease problem in contrast to the higher cost of investing in upgrading the farm environment and improving the management to produce a long-term solution. This concept linked closely with the major theme of ‘agricultural factors’, as management systems were thought to impact on the antimicrobial requirements of farms.

‘*Farmers probably have to accept that medication will not rectify poor husbandry or poor management … Also, yes, antibiotics might be a quick and relatively cheap option to solve a problem on a farm—in terms of cost. But actually, should we be doing something that's a bit more expensive and a bit more long-term? We're sort of depending on short-term, relatively cheaper options.*’

### Knowledge base

Whilst participants showed an awareness of guidelines on the responsible use of antimicrobials in pigs, these were seldom cited as being adopted by veterinary practices. Individual practitioners and practices tended to work independently of these and sought information from their own experience, the history of the farm and colleagues when information beyond an individuals’ experience was required. Mixed species practitioners consulted a wider variety of information sources on antimicrobials and were more likely to seek information from colleagues compared with practitioners working within specialist pig practices.

‘*I mean there already are some guidelines like RUMA, and things like that. But … The trouble is that practices tend to work quite independently … having their individual ways of doing things.*’

‘*We have a practice protocol which would be agreed between the clinicians in the pig department, of which there is now four of us, which would be based on our experience in the past … bearing in mind that there's three of us here with, I don't know, seventy years of experience probably combined of pig practice.*’

Whilst the majority of participants felt that the experience of colleagues was a trusted source of information on antimicrobials, a minority acknowledged that there could be an issue with senior colleagues applying pressure on more junior practitioners, which was often to maintain good relationships between long-term clients and practices. This minority opinion was shared by both junior veterinary surgeons, who identified the pressure, and senior veterinary surgeons who admitted to applying such pressure.

‘*me being a grumpy old partner would have been upset that my young vet cheesed off one of my good old clients …*’

## Discussion

The decision whether to prescribe an antimicrobial, and which antimicrobial to prescribe is influenced by a number of complex factors in the context of pig veterinary medicine. Antimicrobial stewardship initiatives to encourage responsible prescribing behaviours are increasingly common in veterinary medicine, with various UK organizations producing national guidelines on the responsible use in companion and livestock species.^33–36^ However, disease-specific protocols are only published for small animal species^37^ and veterinary guidance is not available at a regional level. Similarly, in human medicine, national prescribing guidelines are widely adopted^38^; however, in contrast, primary care trusts produce and advocate their own local level guidance^39^ targeting specific disease conditions and bacterial pathogens.^40^ Consequently, whilst the decision whether to prescribe an antimicrobial is an individual choice, those working within a human medicine environment have a greater number of targeted information sources to guide such decision-making when compared with veterinary prescribers. Thus, the potential for greater variation between individual prescribing practices by veterinary surgeons, when compared with physicians, highlights this as an area where greater understanding of behavioural influences is needed.

Interviewees highlighted concern over antimicrobial resistance within veterinary and human medicine. Similar concerns and views have been expressed by both companion and farm animal veterinary surgeons.^17,20,41^ Whilst some participants identified that they had encountered resistance in their clinical pig work, many shared the opinion that resistance was an issue faced by other pig practitioners, in other geographic regions and other species sectors. A review of the human literature revealed a widespread awareness by physicians of the issue of antimicrobial resistance in human medicine.^42^ However, in parallel with findings in this study, this concern was often accompanied by a belief that the responsibility for the generation of such resistance lies with other professionals and in other medical establishments.^19,43^

At present there is no integrated collection of data on resistance in zoonotic bacteria within the UK, with human data relying on voluntary laboratory submissions to a national database, whilst veterinary data are obtained through voluntary submissions to the Animal and Plant Health Agency and EU-harmonized monitoring surveillance of isolates from healthy animals.^44^

Whilst the transfer of resistance genes between bacteria from animals to those seen in humans is an accepted phenomenon, the frequency with which this occurs and the level of threat it poses to public health remains unknown.^45,46^ The predominant opinion amongst participants was that this was a sporadic event and that any threat was minimal. Whilst opinions from human prescribers have implicated the overuse of antimicrobials in livestock in human resistance profiles,^42^ it seems veterinary opinion is divided.^16,17,47^ However, there was a shared opinion amongst the study population that antimicrobial resistance in human medicine was mainly driven by use in humans, an opinion echoed in the literature.^48–50^

The desire of the prescriber to maximize compliance has been recognized previously as influencing prescribing decisions by doctors^51,52^ and veterinary surgeons,^17,21^ and was commonly used to justify the choice of a particular antimicrobial by interviewees. Some interviewees described that in some clinical situations they may have chosen a long-acting preparation of a critically important antimicrobial as a first-line option due to concern that a farmer was unlikely to be able to administer repeat injections of an alternative short-acting antimicrobial. Similarly, the benefits of long-acting antimicrobial preparations for improving farmer compliance have been identified as crucial in livestock species in which repeat administration of injectable antimicrobials can be practically difficult.^21,53^ Educational initiatives for producers on the importance of completing a course of antimicrobials, including practical solutions and training for administering repeat injections, may reduce the use of longer-acting critically important antimicrobials.

Disease prevention through antimicrobial prophylaxis is perceived to be prudent by human^54,55^ and veterinary prescribers,^14,16^ although is a much more common phenomena in production animal medicine.^15^ Interview participants described an ethical conflict between responsible prescribing and ensuring that the health and welfare of the pig is maintained. This has been recognized as an issue in the veterinary sector^56^ and in human medicine.^43,57^ Veterinary surgeons acknowledged that deciding to withdraw prophylactic antimicrobials on individual farms was problematic due to farmer reluctance to do so and concern by the veterinary surgeon that signs of disease may return on withdrawal. This was also a concern shared by practitioners attending a Veterinary Medicines Directorate focus group on prescribing pressures who identified the difficulty of preventing certain diseases on farms where infections are endemic.^53^ Further research to investigate feasible alternative methods of preventing disease to the use of antimicrobials for a typical commercial UK pig unit may allow farmers to reduce such use.

The participants described complex and varying relationships between the veterinary surgeon and the farmer. In some situations, participants felt pressure that farmers expected an antimicrobial prescription and in some instances, they faced overt demands from farmers for particular antimicrobials. Client pressure has been identified as an influence on prescribing behaviours in human medicine,^58–60^ and others have shown that client expectation can influence prescribing behaviour in farm animal medicine.^17,21^ In contrast, some participants defined a mutual relationship between the veterinary surgeon and the farmer, whereby prescribing decisions were shared between both parties. In human medicine the importance of maintaining the doctor–patient relationship has been found to have a positive influence on prescribing behaviours^61,62^ whilst Visschers *et al*.^63^ showed the value that pig farmers’ placed on their veterinary surgeon for information on antimicrobials, the risks associated with use and alternatives to such use. These results suggest that effective communication and improved veterinary surgeon–client relationships may be beneficial in increasing farmer awareness of the issues surrounding antimicrobial use and may reduce the issue of client pressure described by some participants.

Fears of being blamed should antimicrobials not be prescribed and later prove to be necessary, and the potential litigation resulting from such a situation, were cited as concerns by veterinary surgeons. Similar fears and pressures have been found to influence prescribing decisions in human medicine^60,62,64^ and veterinary prescription behaviours in livestock species.^17,21^ Another concern identified by some of the junior-level veterinary surgeons was pressure to prescribe from senior colleagues, which was also a concept identified in human medicine.^65^ The ability to mitigate veterinary surgeons fear of diagnostic uncertainty is complex due to the unpredictability of which animals may suffer negative consequences should antimicrobials not be prescribed and then later be required. Greater educational opportunities for veterinary surgeons on the importance of using an evidence-based medicine approach, diagnostic testing to support clinical decisions and the negative consequences from unnecessary prescribing, may alleviate some of these pressures from practitioners. The wider adoption of practice protocols based on collective veterinary surgeon experience, disease profiles from diagnostic submissions or surveillance and existing national guidelines on antimicrobial use in pigs may build confidence and guide more junior veterinary surgeons to make independent prescribing decisions. Participants identified that antimicrobial use often targeted disease syndromes potentially consisting of multiple viral and bacterial pathogens. Similarly, such disease syndromes have been recognized as a potential motivation for antimicrobial prescribing in human^66^ and veterinary medicine.^67,68^ In human medicine it has been shown that co-infection with bacteria in acute viral respiratory disease could worsen the disease clinical signs.^69^ Similarly, participants identified that secondary bacterial infections would result in more advanced clinical signs in pigs if antimicrobials were not used. Thus, controlling and preventing viral disease on pig units is vital to minimizing antimicrobial use.

In chronic disease situations, where a prolonged antimicrobial course is indicated, interviewees expressed a preference for in-feed antimicrobials with the lower cost considered a motivation. A minority of the sample population proposed that the use of in-feed antimicrobials for disease prevention in some circumstances might be used for their beneficial effects on growth rates. Therefore, there was conflict in what participants defined as prudent use, with some considering the use of tylosin to prevent ileitis, caused by *L. intracellularis*, as being responsible use, and others considering this practice to be, in reality, for its growth promotion properties and thus such use was not justified.

Routine diagnostic testing to determine the disease status in a herd was seldom cited by the interviewees. Similarly, it has been found to be infrequently conducted in farm animal veterinary practice^41,53^ and is considered an underutilized tool by physicians.^70,71^ Veterinary surgeons recognized the merits of AST in new disease outbreaks but they highlighted concerns over the time lag in obtaining results if immediate treatment was required. Such concerns are highlighted in this study and are echoed in the human^72^ and veterinary literature.^17,41^ The availability of ‘pen side’ and rapid diagnostic tests, to identify both pathogens and antimicrobial susceptibility profiles, would allow more targeted antimicrobial use and avoid the requirement to change antimicrobials following susceptibility results. In addition, more structured surveillance data on bacterial isolates and susceptibility profiles in animal disease would allow practitioners to make more informed prescribing decisions in the absence of AST results.

Participants described a social responsibility to reserve the use of critically important antimicrobials for cases where these classes are the only therapeutic option; this has also been shown to influence antimicrobial use by human^43,57^ and veterinary prescribers.^41^ However, contradiction was observed within the study as whilst participants described an ideology where fluoroquinolones are a second-line therapeutic choice, their use was commonly cited as being a primary therapeutic in the treatment of piglet scour. This inconsistency between the desired behaviour, to ensure that antimicrobial use is responsible, and the actual prescribing decision is shown by Busani *et al*.^20^ where 54% of veterinary surgeons chose a fluoroquinolone as a first-line choice in a calf scour scenario despite showing a high awareness of judicious antimicrobial use practices. Justifications amongst participants for the use of fluoroquinolones in piglet scour were high resistance levels to other antimicrobials in neonatal bacterial diarrhoea and the lack of availability of non-critically important antimicrobials for these cases. In parallel, fluoroquinolones were shown to be used in 12% of diarrhoeal conditions in pigs in Europe,^73^ and the lack of availability of some antimicrobials was also identified in a veterinary focus group discussion on prescribing pressures in UK pigs.^53^ It is essential that veterinary surgeons regularly perform AST to monitor resistance profiles and ensure that treatment choices for neonatal scour are appropriate and justified. However, this effort needs to be coupled with further research into methods through which neonatal diarrhoea can be prevented and managed within pig units.

Discussion over potential restrictions on the veterinary use of critically important antimicrobials sparked unease amongst participants. Greatest concern in terms of pig health lay with the potential restriction of the macrolides, followed by the fluoroquinolones and the least concern was reported over the third- and fourth-generation cephalosporins. A study examining the frequency of antimicrobial use in European pigs showed that 10.8% of prescriptions were macrolides, 7.2% were fluoroquinolones and 2.3% were third- and fourth-generation cephalosporins.^73^

Maximum residue limits for veterinary medicinal products are solely based on pharmacological data with no consideration of the potential influence on antimicrobial use.^74^ The importance of choosing a product with a suitable withdrawal period was highlighted by interviewees and is an economic factor unique to the food animal sector. For example, the nil withhold period on in-feed tylosin formulations was described as a motivation towards its use in pigs near slaughter. As a key driver towards prescribing behaviours^17,53,75^ the introduction of longer maximum residue limits on tylosin may be beneficial in reducing prescribing in livestock near slaughter weight, which may be a potential intervention to drive more targeted and responsible use of the highest priority, critically important, antimicrobials. However, this perceived benefit would have to be judged against the background of possible health, welfare and productivity impacts that may occur.

Veterinary surgeons’ ability to profit from the sale of antimicrobials has been highlighted as a potential conflict of interest.^76,77^ In reaction to this concern, the European Parliament has debated the possibility of ‘decoupling’ antimicrobial sales to eliminate the potential that profit may drive prescribing behaviours.^78^ However, the impact of such policy in other European countries shows diverse results from countries and hence the impact appears unclear and may be complex and influenced by other factors. A study examining pig farmer perceptions in Switzerland showed that there was a neutral opinion on such a policy^79^ whilst study participants expressed widespread concern that such a move would have a negative impact on the financial stability of practices and on the health and welfare of animals, which are views shared by the UK and European veterinary professions.^80,81^

Despite agreement that environment and good management were important influences on antimicrobial use, participants presented a wide range of views, with no consensual opinion, on which farming systems related to high or low quantity of antimicrobial used. Such diversity of opinion is shown in the literature when the interaction of farming systems with the health and welfare of pigs is deliberated.^82–86^ One distinct viewpoint presented by some interviewees was that straw-based solid flooring was linked with a greater quantity of *Salmonella* spp. than slatted systems. The literature does not support this proposal as a similar prevalence of *Salmonella* spp. has been reported in slaughter pigs raised in both slatted and straw-based systems.^87^ Thus, further research is needed on the association between different housing, flooring and management systems on the incidence of disease, resistance and the use of antimicrobials to identify optimum housing and management to reduce and prevent clinical disease and thus reduce the clinical need for antimicrobials.

Participants identified that having highly skilled staff was essential in ensuring that a unit is well managed, which is a concept echoed in the literature.^88^ In addition, interviewees identified vaccination as an highly desirable alternative to antimicrobial use for pig disease prophylaxis, as reflected in other studies.^16,89,90^ Further evidence of the most efficient systems in terms of reducing antimicrobial use and resistance, whilst maintaining productivity and providing economic return, are not currently available.

Seeking alternatives to antimicrobial use in pigs using an integrated approach that considered the pig within its herd and environment was a concept defined by participants. Ensuring that pigs have an optimal farm environment with high-quality hygiene, biosecurity and management practices have been associated with low antimicrobial use.^86,91^ In parallel with the literature, veterinary surgeons identified that in some cases improved biosecurity and environmental management have been considered as viable alternatives to prescribing by reducing the need for treatment.^89,90^ However, participants felt that the costs of such improvements may be considered prohibitive and reinvestments in farming systems are not feasible due to the high costs involved, which is an opinion echoed in the literature.^17,90,92^ In parallel, whilst the economic and health benefits of MRSA control programmes in human hospitals are clear, human medicine faced similar financial hurdles in implementing effective disease prevention and control programmes in tertiary care settings.^93^ A comprehensive cost–benefit analysis of improving management practices and facilities, and reducing antimicrobial use, in typical UK pig production systems may be beneficial in providing evidence for methods that will optimize the use of antimicrobials.

### Conclusions and implications

This study offers in-depth insights into the complex influences behind antimicrobial prescribing decisions by UK pig veterinary surgeons. Veterinary surgeons were very aware of antimicrobial resistance and identified a social responsibility for prudent use; however, there was a failure to perceive it as relevant to their own situation. Antimicrobial resistance was not considered a major problem for the health of pigs in the UK, despite identification of treatment failures and pathogens that were resistant to some classes of antimicrobials. This combined with other influences, such as a strong responsibility to ensure animal health and welfare, pressure from clients to prescribe and a lack of alternative and economically viable strategies, such as management improvements or investment in new facilities, may all lead to inappropriate prescribing in some contexts. Interventions to optimize or reduce antimicrobial use on farms will need to consider these multiple factors if they are to be successful across the pig industry.

## Funding

The study was funded by a grant awarded to G. L. P. from the Veterinary Medicines Directorate, an executive agency of the Department for Environment, Food and Rural Affairs.

## Transparency declarations

None to declare.

## Supplementary data

Tables S1 and S2 are available as Supplementary data at *JAC* Online (http://jac.oxfordjournals.org/).

Supplementary Data
