# Supplementary material for: Understanding the culture of antimicrobial prescribing in agriculture: a qualitative study of UK pig veterinary surgeons
Source: J Antimicrob Chemother. 2016 Aug 11;71(11):3300–12. doi: 10.1093/jac/dkw300 (PMC5079303; doi:10.1093/jac/dkw300)
Supplement: Supplementary Data [file supp_71_11_3300__index.html]

Understanding the culture of antimicrobial prescribing in agriculture: a qualitative study of UK pig veterinary surgeons — Understanding the culture of antimicrobial prescribing in agriculture: a qualitative study of UK pig veterinary surgeons — Supplementary Data 

# Understanding the culture of antimicrobial prescribing in agriculture: a qualitative study of UK pig veterinary surgeons

## Supplementary Data

Supplementary Data

- Supplementary Data - Docx file
