# Supplementary material for: Understanding the culture of antimicrobial prescribing in agriculture: a qualitative study of UK pig veterinary surgeons
Source: J Antimicrob Chemother. 2016 Aug 11;71(11):3300–12. doi: 10.1093/jac/dkw300 (PMC5079303; doi:10.1093/jac/dkw300)
Supplement: Supplementary Data [file supp_dkw300_dkw300supp.docx]

**Supplementary data**

**Table S1.** Demographic data for participants

| **Demographic information** | **Category** | **n** |
| --- | --- | --- |
| **Gender** | Female | 4 |
|  | Male | 17 |
| **Company or Practice** | Practice | 19 |
|  | Company | 2 |
| **Pig density of geographic location** | High | 11 |
|  | Moderate | 7 |
|  | Low | 1 |
|  | UK-wide^2^ | 2 |
| **Type of work** | Farm only | 2 |
|  | Mixed | 9 |
|  | Pig only | 10 |
|  | Assistant | 6 |
| **Position in practice** | Partner | 13 |
|  | Consultant^1^ | 2 |
| **Years since graduation** | 0-5 years | 3 |
|  | 6-10 years | 1 |
|  | 11-15 years | 0 |
|  | 16-20 years | 3 |
|  | 21-25 years | 3 |
|  | >26 years | 11 |
| **Postgraduate study** | DBR | 1 |
|  | DipECPHM | 1 |
|  | CertPM | 3 |
| **Breeding sows under care of the veterinary surgeon** | Less than 999 | 4 |
|  | 1000-5000 | 8 |
|  | 5001-10,000 | 3 |
|  | More than 10,001 | 2 |
|  | No information | 4 |
| **Finishing pigs under the care of the veterinary surgeon^3^** | Less than 50,000 | 8 |
|  | 50,001-100,000 | 1 |
|  | 100,001-200,000  200 | 1 |
|  | More than 200,001 | 2 |
|  | No information | 9 |
| **Caseload - indoor/outdoor split** | All indoor | 9 |
|  | Majority indoor | 6 |
|  | Majority outdoor | 5 |
|  | No information | 1 |

^1^ - Consultant – Advisory role, does not prescribe drugs

^2^ - Consultant works throughout the UK

*^3^* – If respondent stated the number of finishing pigs was progeny from breeding sows on a breeding to finishing farm an estimate of the number of finishing pigs was calculated based on the UK average of 25.8 pigs finished per breeding sows per year (Source - AHDB Pork)

*Cert PM – Certificate in Pig Medicine, DipECPHM - European Diploma from the European College of Porcine Health Management (ECPHM)

**Table S2.** Intrinsic and extrinsic factors identified as influencing antimicrobial prescribing behaviours through in-depth qualitative interviews with 21 veterinary surgeons working in pig practice in the UK

| **Factors intrinsic to the prescribing veterinary surgeon** | **Factors extrinsic to the prescribing veterinary surgeon** |
| --- | --- |
| **Knowledge Base**   1. Colleagues 2. NOAH Compendium 3. Own experience 4. Pharmaceutical companies 5. Pig Veterinary Society 6. Scientific information sources   **Responsibility**   1. Personal prudent prescribing principals 2. Prudent prescribing by other veterinary surgeons 3. Non-prudent prescribing by other veterinary surgeons 4. Prudent antimicrobial use by farmers 5. Non-prudent antimicrobial use by farmers 6. Professional responsibility to health and welfare of pig 7. Compliance   **Veterinary Surgeon-Client Relationship**   1. Mutual relationship 2. Client pressure | **Agricultural Factors**   1. Farming system  - Pig flow (all-in-all-out, continuous) - Housing type (indoor, outdoor, slatted, straw)  1. Management of unit 2. Biosecurity 3. Health status 4. Farm environment 5. Quality of stockmanship 6. Geographic farm location 7. Genetics of pig herd 8. Weather   **Disease Epidemiology and Outcomes**   1. Dynamic nature of disease  - Acute disease - Chronic disease  1. Increased mortality rates 2. Diagnostic testing  - Routine diagnostic testing - Diagnostic testing in a novel disease situation  1. National disease surveillance 2. Alternative methods of disease prevention to antimicrobial use   **Drug-related Factors**   1. Availability of antimicrobial 2. Good efficacy 3. Poor efficacy  - Antimicrobial resistance - Treatment failure  1. Withdrawal periods 2. Type/class of antimicrobial 3. ‘Critical’ antimicrobials 4. Formulation of antimicrobial 5. Antimicrobial use for disease prevention   **Economic Factors**   1. Veterinary practice as a business 2. Profit from medicine sales for veterinary practices 3. Competition from neighbouring veterinary practices 4. Pig farm as a business 5. Veterinary costs for farm   **External Pressures**   1. Media pressure 2. Public perception pressure 3. Fear of litigation 4. Regulatory and political pressure 5. Retailer pressure 6. Importation pressure 7. High antimicrobial use abroad 8. High antimicrobial use in other veterinary sectors 9. High antimicrobial use in human medicine |
